# The effects of polymorphisms on human gene targeting

**DOI:** 10.1093/nar/gkt1303

**Published:** 2013-12-25

**Authors:** David R. Deyle, Li B. Li, Gaoying Ren, David W. Russell

**Affiliations:** ^1^Department of Medicine, University of Washington, Seattle, WA, 98195, USA and ^2^Department of Biochemistry, University of Washington, Seattle, WA, 98195, USA

## Abstract

DNA mismatches that occur between vector homology arms and chromosomal target sequences reduce gene targeting frequencies in several species; however, this has not been reported in human cells. Here we demonstrate that even a single mismatched base pair can significantly decrease human gene targeting frequencies. In addition, we show that homology arm polymorphisms can be used to direct allele-specific targeting or to improve unfavorable vector designs that introduce deletions.

## INTRODUCTION

Gene targeting has revolutionized genetics and allowed for site-specific manipulation of the mammalian genome. Increasingly, it is now used to engineer human cell lines and it may be used therapeutically in the future. The human genome contains multiple types of genetic variations, with single-nucleotide polymorphisms (SNPs) occurring on average every 1000–2000 bp when comparing haplotypes (1). Because the homology arms present in targeting vectors are frequently several killobases in length, these polymorphisms could impact homologous pairing and recombination.

Prior studies in mouse embryonic stem cells showed that gene targeting frequencies were lower when vectors contained as few as 0.6% DNA mismatches, which has led to the routine use of isogenic DNA for the preparation of targeting constructs (2–4). Surprisingly, in the case of human cells, a comparison of targeting frequencies at eight different loci in seven human cell lines concluded that isogenic DNA was not advantageous and that human recombination was tolerant of mismatches (5). However, in these human cell experiments, it was not established if the targeting constructs included polymorphisms not present in the chromosomal target loci. Given the importance of sequence homology on recombination in other systems, we examined this issue again in human cells. Here we show that the number and position of SNPs affect gene targeting, and that the inclusion of polymorphisms in vectors can enhance targeting at a specific allele and the generation of a deletion mutation.

## MATERIALS AND METHODS

### Plasmids and vectors

The plasmids pA2HSN5′ (6), pA2-APPe3ITKNA (7), pA2HPe3 (8) and pHPe2/3 (8) have been described. Plasmids pLHSNΔ53O and pLHSNins4O are based on pLHSNO (9), but contain a 53-bp deletion at base pair 63 or a 4-bp insertion at base pair 64 of the *neo* reading frame, respectively. Plasmid pLHSNΔ53O-SNP6 is identical to pLHSNΔ53O except that G to A transitions were introduced by sequential site-directed mutagenesis into the 5′ homology arm at −2, −87, −304, −700, −999, −1503 bp relative to the *neo* translation start site. Plasmids pLHSNΔ53O−2; pLHSNΔ53O−87, −304, −700; pLHSNΔ53O−999, and −1503; and pLHSNΔ53O−1503 contain only the specified SNPs. Plasmids pLHSΔ1Nins4O, pLHSΔ4Nins4O, pLHSΔ1Nins4Δ1O and pLHSΔ4Nins4Δ4O are identical to pLHSNins4O except that 1 or 4 bp deletions were engineered at base pair −5 and +103 relative to the *neo* start site. Plasmids pA2HPe3(i2i3+1) and pHPe2/3(i2i3+1) are identical to pA2HPe3 and pHPe2/3, respectively, except for 1 bp insertions located 15 bp upstream and 20 bp downstream of *HPRT* exon 3. All plasmid sequences are available on request.

Retroviral vectors were produced by calcium phosphate transfection of PG13 packaging cells (10) with pLHSNΔ53O, pLHSNins4O or their derivatives, collection of medium 2 days later and passage through 0.45-µm filters. Adeno-associated virus (AAV) vectors AAV-HSN5′, AAV-APPe3ITKNA, AAV-HPe3 and AAV-HPe3(i2i3+1) were prepared by transient cotransfection of helper and vector plasmids as described (11). All AAV vector stocks were serotype 2, purified on iodixanol gradients, and titered by quantifying full-length vector genomes on alkaline Southern blots (12). To ensure that an equivalent number of AAV genomes were used to transduce HT-1080 subclones in the *HPRT* experiments, AAV-HPe3 and AAV-HPe3(i2i3+1) titers were determined on the same alkaline Southern blot.

### Cell culture

HT-1080 human fibrosarcoma cells (13) were cultured at 37°C in Dulbecco's modified Eagle's medium with 10% heat-inactivated fetal bovine serum (Hyclone), 100 U/ml of penicillin and 100 µg/ml streptomycin. Moloney murine leukemia virus (MLV) provirus target sites were introduced into HT-1080 cells and selected with hygromycin to produce polyclonal transduced populations derived from >10^4^ independent transduction events, as determined by plating dilutions of the transduced cells in selective medium as described (9). Down syndrome induced pluripotent stem cells (iPSCs) were derived from Down syndrome fibroblasts (AG06872; Coriell Institute for Medical Research) as described previously (7). iPSCs were grown on irradiated mouse embryonic fibroblasts as described (14).

### Gene targeting

SNPs were engineered into the MLV target sites rather than the AAV targeting vectors to allow for the rapid generation of genetic variants and the use of a single gene targeting vector to minimize experimental variation. AAV-HSN5′ was used to correct *neo* mutations as described (9). *APP* locus gene targeting was conducted as described (7). In Figure 4, independent HT-1080 subclones Δ4 c1 and Δ4 c2 were engineered to contain a 4-bp deletion in exon 3 of *HPRT*, and independent subclones +4 c1 and +4 c2 harbor a 4-bp insertion in exon 3 of *HPRT* as previously described (8). When correcting *HPRT* mutations with AAV vectors, HT-1080 *HPRT* subclones were plated at 5.5 x 10^5^ cells per 6 cm dish on day 1 and infected with AAV-HPe3 or AAV-HPe3(i2i3+1) at an multiplicity of infection of 10^4^ genome-containing particles on day 2. On day 3, cells were treated with trypsin and replated at 0.008 and 99% dilutions in 10-cm dishes and 15-cm dishes, respectively. On day 4, the cells in 99% dishes were grown in HAT medium (contains hypoxanthine, aminopterin, and thymidine) and the 0.008% dishes were grown without selection. After 7–10 days, colonies were counted. Gene targeting with transfected linearized plasmids pHPe2/3 and pHPe2/3(i2i3+1) was done by transfecting with Superfect reagent (Qiagen) as described (8). Briefly, HT-1080 subclones with *HPRT* mutations were plated at 5 x 10^5^ cells per 10-cm dish (42 dishes for each subclone) on day 1, and 20 dishes were each transfected with 10 µg of linearized pHPe2/3 and 0.1 µg of pCMVβ, or 10 µg of linearized pHPe2/3(i2i3+1) and 0.1 µg of pCMVβ on day 2. pCMVβ, which expresses the *lacZ* gene from a cytomegalovirus (CMV) promoter (Clontech) was used as a transfection efficiency control. On day 3, the transfected cells were treated with trypsin and pooled, 5 × 10^4^ cells were plated in a well of a 6-well plate for β-galactosidase staining, and the remaining cells were counted and plated in 20 15-cm dishes. On day 4, the six-well plate was stained for β-galactosidase expression and the number of positive foci determined. On day 5, the remaining dishes were switched to HAT medium for selection, and the surviving colonies were counted 10–14 days later.

### DNA analysis and plasmid rescue

Genomic DNA was isolated by the Puregene DNA purification protocol (Gentra Systems/Qiagen). To determine whether mutations were introduced into the target loci during retroviral production or cell expansion, genomic DNA was isolated from HT-1080 polyclonal populations containing LHSNΔ53O and LHSNΔ53O-SNP6 and the MLV sequences homologous to AAV-HSN5′ were amplified by polymerase chain reaction (PCR) using primers LHSNO-for (ACCTGAGGAAGGGAGTCGAT) and LHSNO-rev (CGCTATGTCCTGATAGCGGT). Ten independent PCR products for both LHSNΔ53O and LHSNΔ53O-SNP6 were cloned into the TA cloning vector pGEM T-easy (Promega), sequenced and all were found to lack mutations. Integrated MLV provirus target loci were rescued as described (15) with the following modifications: 20 µg of genomic DNA containing a corrected MLV site was digested with 80 units of Kpn I, extracted with phenol and chloroform and precipitated with ethanol. The resulting DNA fragments were resuspended and circularized with 2000 U of T4 DNA ligase in 400 µl at 16°C overnight. The DNA was precipitated, resuspended in 10 µl of H_2_O and 2 µg was electroporated into supercompetent *Escherichia coli* DH10B cells (Invitrogen). Targeted *APP* alleles were determined by PCR amplification of the 5′ homology region as described (7).

### Statistical analysis

In all cases, statistical significance was determined using Student’s *t*-test to compare gene targeting frequencies. *P* < 0.05 were considered significant.

## RESULTS

### Homology arm SNPs influence gene targeting frequencies

We designed a SNP-dependent targeting assay in which a MLV vector that confers hygromycin resistance is used to deliver a mutated *neomycin phosphotransferase* (*neo*) gene, which is then corrected by an AAV gene targeting vector to generate G418-resistant colonies. AAV-mediated gene targeting is efficient in human cells (16), so targeting frequencies can be measured accurately with this system. The MLV vectors LHSNΔ53O and LHSNΔ53O-SNP6 used to deliver the target loci both have a 53-bp deletion in the *neo* gene, but LHSNΔ53O-SNP6 also contains six G to A SNPs in the 5′ homology arm located at −2, −87, −304, −700, −999 and −1503 bp relative to the *neo* start site. The targeting vector AAV-HSN5′ contains 3149 bp of sequence homology to LHSNΔ53O, with a truncated *neo* gene that lacks the 53 bp deletion.

**Figure 1.**
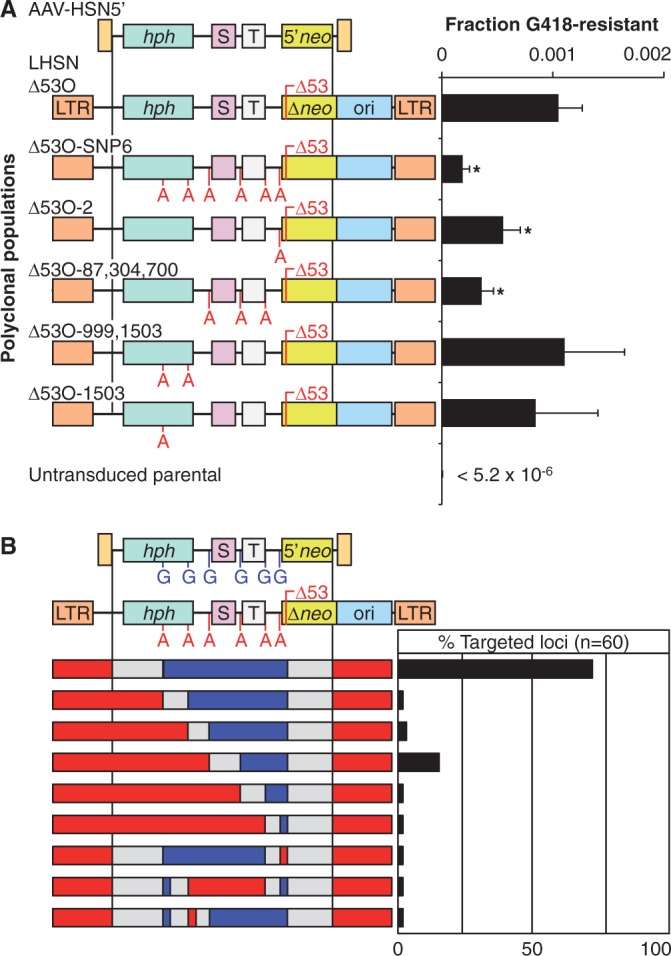
Gene targeting at MLV target loci with SNPs. (**A**) Schematic of the AAV-HSN5′ targeting vector and six MLV provirus loci with the location of the 53 bp *neo* gene deletion (Δ53) and the G to A SNPs shown. Targeting frequencies were measured as the fraction of G418-resistant colonies obtained after infecting polyclonal HT-1080 populations containing each indicated MLV provirus with AAV-HSN5′. LTR, long terminal repeat; *hph*, hygromycin phosphotransferase; S, SV40 promoter; T, Tn5 bacterial promoter, 5′*neo*, truncated neo gene; ori, p15A plasmid origin; *P < 0.05 versus LHSNΔ53O. (**B**) Graphic illustration of SNPs present in the recovered targeted loci. Colors indicate the presence of MLV target SNPs (red), AAV vector SNPs (blue) or segments between identifiable SNPs (gray).

HT-1080 human fibrosarcoma cells were transduced with LHSNΔ53O or LHSNΔ53O-SNP6 to generate polyclonal populations consisting of at least 10^4^ independent target proviruses. This allowed us to avoid genomic position effects, which can influence targeting frequencies significantly (6). To ensure the fidelity of MLV target loci in these polyclonal populations, integrated proviral DNA was amplified by PCR and the region homologous to the AAV targeting vector was sequenced. We analyzed 10 independent PCR products from each polyclonal population and did not identify any mutations. Both populations were infected with AAV-HSN5′ and the number of G418-resistant colonies determined (Figure 1A). The six SNPs present in LHSNΔ53O-SNP6 decreased targeting ∼5-fold. Next, we introduced additional MLV vectors with different combinations of SNPs in the homology arm region to determine the effects of SNP position and number on gene targeting (Figure 1A). A single SNP reduced targeting when it was present 64 bp from the *neo* deletion (LHSNΔ53O−2 target), but SNPs located ∼1 kb or more from the deletion had little or no measurable effect (LHSNΔ53O−999,1503, for example). In general, the decrease in targeting frequencies due to SNP mismatches was greatest when they were closest to the mutation being corrected, and the effect of multiple SNPs was additive. Additional statistical comparisons between each SNP pattern are shown in Supplemental Figure S1.

### Mapping the extent of chromosomal sequence changes

We used this system to determine the extent of vector homology arm sequence introduced into the human genome during gene targeting by tracking which SNPs were present in targeted loci. The LHSNΔ53O-SNP6 vector contains a bacterial promoter and replication origin that allows for the rescue of integrated proviruses as circularized bacterial plasmids. Sixty independent gene-targeted clones were isolated, and their LHSNΔ53O-SNP6 target loci were rescued from genomic DNA and sequenced. Based on the presence of SNPs, 72% of targeting events incorporated all of the identifiable vector homology arm sequence into the chromosome, extending to the distal 1016 bp of the 5′ homology arm over 1.5 kb from the 53-bp deletion mutation (Figure 1B). This could be due to preferential recombination near the vector inverted terminal repeats, which may form a recombinogenic hairpin structure (17), or extended tracts of mismatch repair occurring on vector:chromosome heteroduplexes. The next most frequent region that limited the extent of vector SNP incorporation during targeting was in the SV40 promoter located 366–762 bp from the *neo* deletion, which could be due to genomic instability associated with these sequences (18). Three clones had a discontinuous SNP pattern that could not be explained by a single recombination or gene conversion event. In these cases, segmented DNA mismatch repair could have led to incomplete inheritance of the SNPs as a contiguous block, or there may have been four recombination crossover events to account for the discontinuity. None of the targeted loci corrected the *neo* mutation without an accompanying A to G conversion.

### SNPs affect gene targeting at an endogenous locus

**Figure 2.**
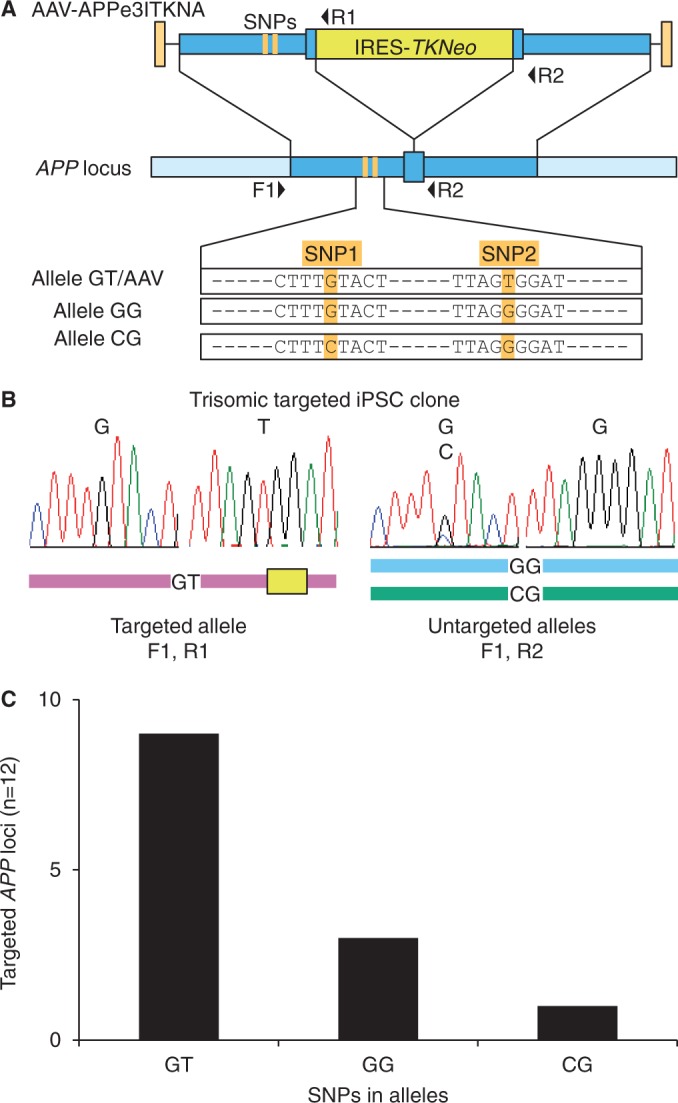
Targeting in trisomic iPSC cells containing three different SNP haplotypes. (**A**) *APP* locus showing AAV-APPe3ITKNA targeting vector, forward (F1) and reverse (R1, R2) PCR primers, SNP locations and sequences found in each of three *APP* alleles. (**B**) Representative sequence reads are shown demonstrating targeting at the GT allele in a Down syndrome iPSC clone. (**C**) The percentage of targeted *APP* alleles with each SNP haplotype.

Based on these results, we reasoned that SNPs should also impact targeting at an endogenous chromosomal locus. The amyloid precursor protein (*APP*) gene on chromosome 21 contains two SNPs located 339- and 389-bp 5′ of exon 3. We transduced a Down syndrome–iPSC line harboring three different *APP* SNP haplotypes (GT, GG and CG) with an AAV targeting vector that contained the GT SNPs in the 5′ homology arm, and was designed to introduce an internal ribosome entry site (IRES) and *TKNeo* gene into exon 3 (7) (Figure 2A). Twelve G418-resistant, targeted clones were isolated, and the targeted and untargeted alleles were amplified by PCR and sequenced to determine which allele was targeted (Figure 2B). The allele lacking SNP mismatches was targeted in nine of the clones, while those with one and two mismatches were targeted in two and one clones, respectively (Figure 2C). These data corroborate our MLV provirus experiments, and they show that SNPs can also decrease targeting at a chromosomal locus in a human stem cell.

### Insertion polymorphisms can increase gene targeting with deletion vectors

**Figure 3.**
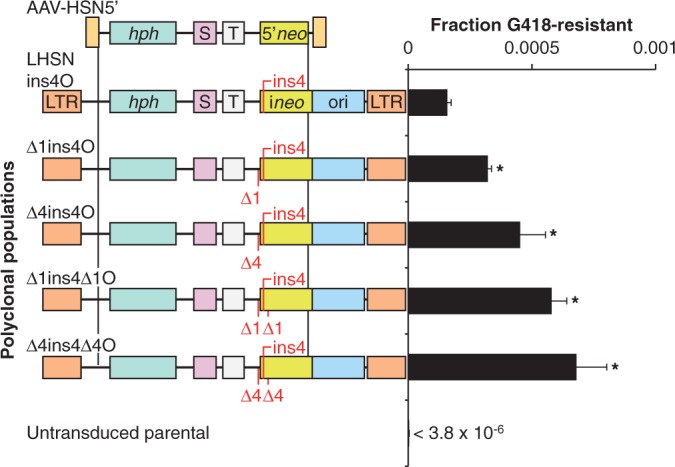
Gene targeting with a deletion vector. Illustration of MLV provirus targets with a 4-bp *neo* insertion (ins4) and different flanking deletions (Δ1, Δ4), with their targeting frequencies as shown in Figure 1. *P < 0.05 versus LHSNins4O.

**Figure 4.**
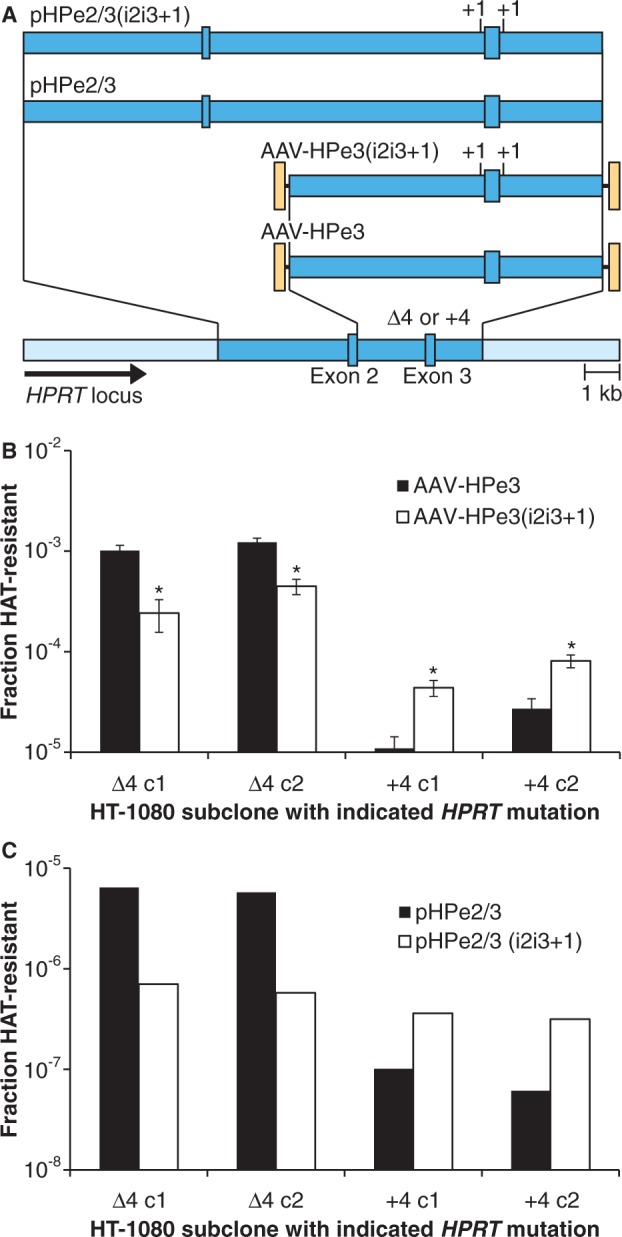
Correction of *HPRT* mutations with AAV and plasmid vectors. (**A**) Structure of the *HPRT* locus containing either a 4-bp deletion or insertion in exon 3, the AAV-HPe3 and AAV-Hpe3(i2i3+1) targeting vectors used and the analogous plasmid-based targeting constructs pHPe2/3 and pHPe2/3(i2i3+1). Gene targeting frequencies are shown as the fraction of HAT-resistant colonies obtained after transducing HT-1080 subclones harboring *HPRT* mutations with either AAV targeting vectors (*P < 0.05 versus AAV-HPe3) (**B**) or linearized plasmid targeting constructs (**C**).

We previously found that introducing an insertion mutation by gene targeting was ∼10 times more efficient than introducing a deletion, suggesting that cellular DNA repair and recombination mechanisms preferentially preserve the unpaired vector insertions that arise in vector:chromosome heteroduplexes (8). We reasoned that insertion polymorphisms in the vector homology arms might increase targeting by a related mechanism. To test this, we redesigned the MLV-LHSNO provirus targeting system so that a 4-bp *neo* gene insertion would be corrected by introducing an unfavorable deletion with the AAV targeting vector. When this target backbone was specifically modified to include 1- or 4-bp silent flanking deletions that could be corrected by favorable insertions in the AAV vector, targeting frequencies increased 4-fold, with the best results obtained when 4-bp insertions were present on both sides of the *neo* mutation (Figure 3). Similar results were obtained when correcting mutations in the endogenous X-linked *HPRT* locus. HT-1080 subclones engineered to contain a 4-bp deletion in exon 3 of *HPRT* (8) were corrected by the AAV-HPe3 targeting vector containing wild-type sequence at 45–93 times the frequency of subclones containing a 4-bp insertion in *HPRT*. In comparison, the AAV-HPe3(i2i3+1) targeting vector containing silent 1-bp insertions in the flanking introns increased targeting 3- to 4-fold in the subclones with an unfavorable insertion in exon 3 (Figure 4A and B).

Silent insertions also increased targeting when transfecting plasmid-based targeting constructs (Figure 4C), demonstrating that the effects of sequence polymorphisms on gene targeting are not limited to AAV vectors. Although we did not calculate statistical significance in these experiments, the results were consistent in two pairs of clones.

## DISCUSSION

These experiments show that DNA polymorphisms have a significant impact on human gene targeting, as demonstrated at multiple target loci in normal and transformed human cells. Similar results were obtained when using single-stranded AAV gene targeting vectors or double-stranded plasmid constructs. Based on these findings, human targeting vectors should contain isogenic DNA to maximize targeting frequencies, as even a single polymorphism can significantly reduce targeting. This can complicate vector design and require the construction of multiple vector stocks, especially when preparing for clinical gene targeting applications in genetically diverse human populations.

Our results show that human cells are like mouse cells, where sequence polymorphisms reduce homologous recombination frequencies (2–4). This result stands in contrast to a prior analysis of human gene targeting (5), which concluded that isogenic DNA did not enhance targeting. However, this prior study did not sequence the target loci to demonstrate if chromosomal SNPs were also present in the plasmid-based targeting constructs, so there may not have been any sequence heterologies. In addition, we found that sequence polymorphism effects were maximal when they were located close to the sequence change being introduced, which may not be the case with many targeting constructs.

We focused on AAV vectors because their high targeting frequencies resulted in many targeted clones and accurate measurements of SNP effects. As shown in Figure 4, the targeting frequencies of transfected plasmid-based constructs were 100- to 1000-fold lower than those of AAV vectors, raising the possibility that unique features of AAV may limit the general applicability of our results. One major difference is that the single-stranded form of the AAV vector genome appears to be the substrate for targeting. This is supported by the lack of targeting observed with AAV vectors containing double-stranded encapsidated genomes (19), and by strand-specific differences in targeting frequencies obtained with related parvoviral vectors that package only one vector strand (20). The AAV capsid could also influence targeting, as shown by microinjection experiments demonstrating that purified AAV vector genomes do not target efficiently (21). Despite these differences, AAV and plasmid-based targeting also have similarities, including stimulation by double-strand breaks (22,23), the involvement of similar recombination proteins (24) and a preference for introducing insertions over deletions (8). Here we show that AAV and plasmid-based targeting are both inhibited by homology arm sequence heterologies, suggesting another shared aspect of their mechanism. Because homologous recombination requires that the plasmid constructs unwind and form heteroduplexes with the chromosome, there is still the opportunity for mismatched bases to reduce homologous pairing or influence mismatch repair, just as with single-stranded AAV vector genomes.

Our results also show that SNPs can be advantageous in certain situations. They can be used to direct allele-specific targeting, which could be useful when correcting distinct mutations in compound heterozygotes, or when inactivating dominant mutations in a single allele, or to avoid recombination with a previously targeted allele when knocking out multiple alleles. Another application of vector sequence heterologies is their potential for enhancing the introduction of unfavorable deletion mutations (8). Although the basis for the reduced targeting frequencies of deletion vectors is not understood, this effect can be minimized by including favorable, silent, flanking insertions in the homology arms.

## SUPPLEMENTARY DATA

Supplementary Data are available at NAR Online

## FUNDING

Funding for open access charge: National Institutes of Health [AR53917 and AR53917-02S1 to D.D.; DK55759, HL53750 and AR48328 to D.R.].

*Conflict of interest statement*. None declared.

## Supplementary Material

Supplementary Data
