# Supplementary material for: The effects of polymorphisms on human gene targeting
Source: Nucleic Acids Res. 2013 Dec 25;42(5):3119–24. doi: 10.1093/nar/gkt1303 (PMC3950700; doi:10.1093/nar/gkt1303)
Supplement: Supplementary Data [file supp_42_5_3119__index.html]

The effects of polymorphisms on human gene targeting — The effects of polymorphisms on human gene targeting — Supplementary Data 

# The effects of polymorphisms on human gene targeting

## Supplementary Data

files

**Files in this Data Supplement:**

- Supplementary Data - pdf file
