# Supplementary material for: The effects of polymorphisms on human gene targeting
Source: Nucleic Acids Res. 2013 Dec 25;42(5):3119–24. doi: 10.1093/nar/gkt1303 (PMC3950700; doi:10.1093/nar/gkt1303)
Supplement: Supplementary Data [file supp_gkt1303_nar-02560-h-2013-File006.pdf]

Supplemental Figure-1 Deyle et al

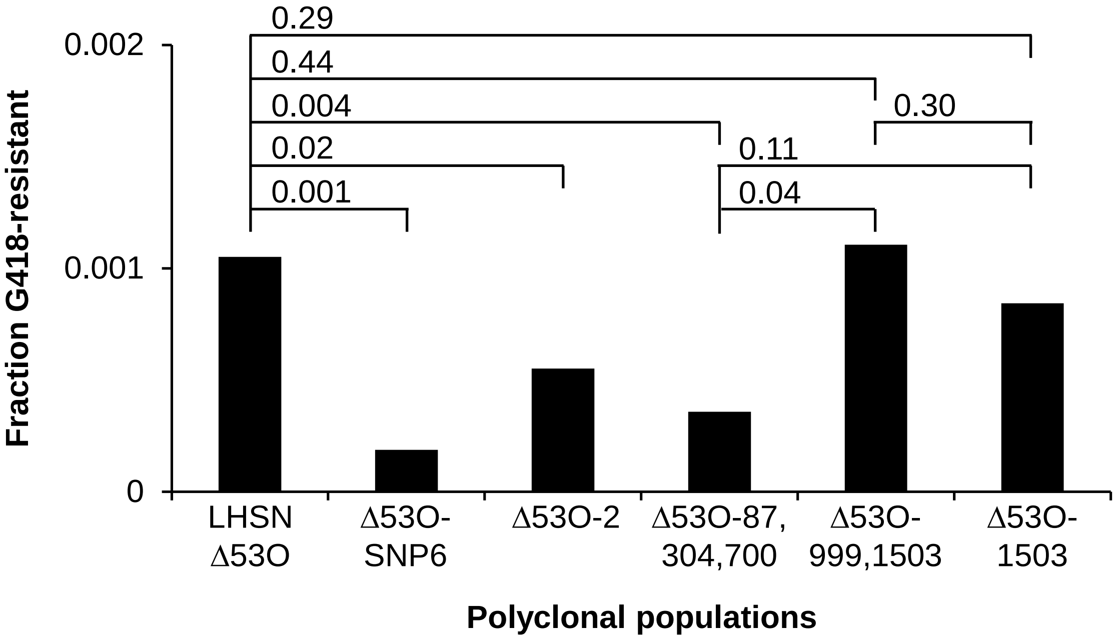

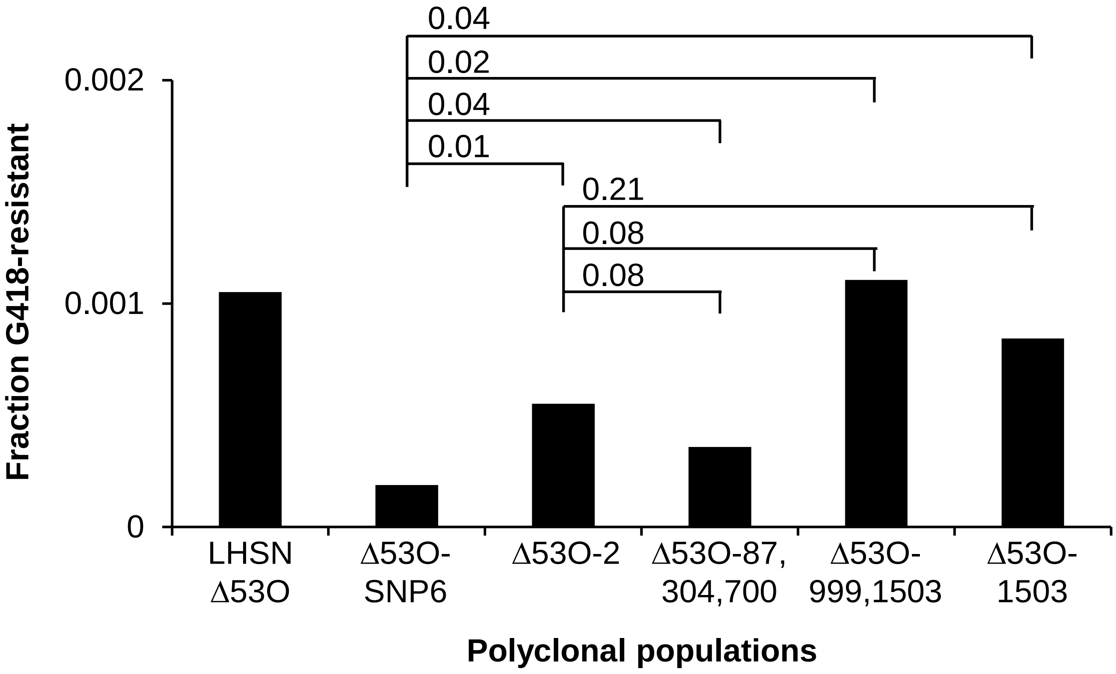

Supplemental Figure 1. Comparison of Gene Targeting Frequencies. Gene targeting frequencies describe in Figure 1. Comparisons of targeting frequencies between different polyclonal HT-1080 populations were analyzed by Student’s t-test and are shown with indicated *P* values.
